# P18 Understanding blood culture sampling practices for adult acute care hospital patients with suspected severe infection: preliminary findings from an ethnographic study

**DOI:** 10.1093/jacamr/dlaf118.025

**Published:** 2025-07-14

**Authors:** Deborah Bamber, Caroline Cupit, Tony Locke, David Jenkins, Tim Coats, Nick Fahy, Alison Prendiville, Laura Shallcross, Eva Krockow, Carolyn Tarrant

**Affiliations:** Department of Population Health Sciences, University of Leicester, Leicester, LE1 7RH, UK; Department of Population Health Sciences, University of Leicester, Leicester, LE1 7RH, UK; PPIE representative, UK; University Hospitals of Leicester NHS Trust, Leicester, LE1 5WW, UK; Department of Population Health Sciences, University of Leicester, Leicester, LE1 7RH, UK; University Hospitals of Leicester NHS Trust, Leicester, LE1 5WW, UK; RAND Europe, Cambridge, CB2 8BF, UK; School of Design, University of the Arts London, London, SE1 6SB, UK; Institute of Health Informatics, University College London, London, WC1E 6BT, UK; School of Psychology and Vision Sciences, University of Leicester, Leicester, LE1 7RH, UK; Department of Population Health Sciences, University of Leicester, Leicester, LE1 7RH, UK

## Abstract

**Background:**

Blood cultures (BC) are vital for early diagnosis and clinical decision-making for patients with suspected infection. However, they are not always taken when antibiotics are started, and suboptimal practices exist that can substantially reduce the quality and usefulness of results. This study aims use routine hospital data, ethnography, and co-design, to understand challenges to reliable BC sampling in hospitals, and to co-develop interventions to improve practice. This poster will focus on the preliminary findings from the ethnographic study.

**Methods:**

Ethnographic methods are being used to explore BC sampling practices for emergency medical patients in three English hospitals. This involves around 150 h of observations in emergency departments and microbiology laboratories. Observations are being captured through written fieldnotes and audio-recorded summaries; key documents and guidelines have also been collected. Up to 45 interviews are being conducted with staff in a range of roles within the three participating hospitals. A first round of observations is complete, with staff interviews underway. A second round of observations will be completed in April 2025. Data are being analysed thematically, drawing on the constant comparative approach.

**Results:**

Preliminary findings focus on descriptions of the everyday practice of managing patients who present to hospitals with suspected infection, and factors shaping BC sampling. Decision-making in emergency departments about whether BCs should be ordered is shaped by guidelines and scoring tools, technology, and local norms. Where there is uncertainty, staff use their clinical judgement to assess patient eligibility for BC sampling. The pathway of decision-making, sample ordering, and sample collection is fragmented and requires coordination and oversight, which can be challenging in a complex and busy environment. The taking of BC sampling may be de-prioritized in situations of high demand, particularly for patients who are severely unwell or difficult to cannulate. Collection of high-quality samples can be compromised by lack of staff training, disorganized equipment, and restrictions within the environment. For ED staff, BC sampling is an isolated event: staff have limited opportunity to see the outcomes of their practice, or to gain insight into the wider value of BC results.

**Conclusions:**

This study has captured the challenges in everyday practice for reliable and high-quality BC sampling. The findings will be used to co-develop interventions to improve practice.

